# Osteotome-Mediated Sinus Lift without Grafting Material: A Review of Literature and a Technique Proposal

**DOI:** 10.1155/2012/849093

**Published:** 2012-06-27

**Authors:** Silvio Taschieri, Stefano Corbella, Massimo Saita, Igor Tsesis, Massimo Del Fabbro

**Affiliations:** ^1^Centre for Research in Oral Health, Department of Biomedical, Surgical and Dental Sciences, Università degli Studi di Milano, IRCCS Istituto Ortopedico Galeazzi, Milan, Italy; ^2^Centre for Research in Oral Implantology, Department of Biomedical, Surgical and Dental Sciences, Università degli Studi di Milano, IRCCS Istituto Ortopedico Galeazzi, Milan, Italy; ^3^Section of Endodontology, The Maurice and Gabriela Goldschleger School of Dental Medicine, Tel Aviv University, Israel

## Abstract

Implant rehabilitation of the edentulous posterior maxilla may be a challenging procedure in the presence of insufficient bone volume for implant placement. Maxillary sinus augmentation with or without using grafting materials aims to provide adequate bone volume. The aim of the present study was to systematically review the existing literature on transalveolar maxillary sinus augmentation without grafting materials and to propose and describe an osteotome-mediated approach in postextraction sites in combination with platelet derivative. The systematic review showed that high implant survival rate (more than 96% after 5 years) can be achieved even without grafting the site, with a low rate of complications. Available alveolar bone height before surgery was not correlated to survival rate. In the described case report, three implants were placed in posterior maxilla after extraction of two teeth. An osteotome-mediated sinus lifting technique was performed with the use of platelet derivative (PRGF); a synthetic bone substitute was used to fill the gaps between implant and socket walls. No complications occurred, and implants were successfully in site after 1 year from prosthetic loading. The presented technique might represent a viable alternative for the treatment of edentulous posterior maxilla with atrophy of the alveolar bone though it needs to be validated by studies with a large sample size.

## 1. Introduction

Implant placement in the posterior maxilla is a challenging procedure when residual bone height is reduced. Maxillary sinus elevation technique is a common surgical procedure which allows to augment the available bone volume in posterior maxilla in order to place implants.

Residual bone height is considered fundamental in deciding which augmentation technique can be used to obtain an adequate bone volume. Generally, sinus lifting through a lateral approach is a viable technique when less than 4-5 mm of residual bone height is present [1–3]. When more than 5 mm of residual bone height is available, a transalveolar approach could be indicated in order to reduce the morbidity and the invasivity of the treatment protocol [4–6].

Osteotome-mediated transcrestal sinus lift approach was first proposed by Tatum in 1986 [7]. In the original approach, implants were placed after the controlled fracture of sinus floor and were submerged during the healing phase. In 1994, Summers described a modification of this technique [8]. The author proposed the preparation of implant site through the use of conical osteotomes which allows the compression, through lateral force application, of the bone in the posterior maxilla. The author stated that these maneuvers allow to increase the lateral bone density, preserving bone because drilling is avoided.

While the transcrestal approach is considered more conservative than the lateral approach, the main drawback is that the sinus lifting procedure must be performed blindly because of the impossibility to visualize the sinus floor [5, 6]. In spite of this limitation, membrane perforation was reported to be less frequent in the osteotome-mediated procedure [6] than in the lateral approach, for which such complication was described in 25–44% of cases [9–11]. 

Transcrestal, osteotome-mediated sinus lift surgery may be performed with or without the use of bone grafting material as allograft, autogenous bone, or heterologous bone material [6]. No significant differences in terms of implant survival and success rates were observed comparing the two methods [6]. Also, the use of platelet derivatives without any bone substitute is described in literature [12, 13] with the aim of allowing a better control of forces during sinus floor elevation and reducing the incidence of complications.

The aim of this study was to perform a literature review regarding osteotome-mediated sinus lifting without bone grafting material and to present a technique to perform the procedure with the use of plasma rich in growth factors (PRGFs).

## 2. Literature Review

### 2.1. Materials and Methods

An electronic search was conducted via MEDLINE (PubMed) in the dental literature to select human clinical trials published from 1986 to January 2012. The search terms used were “sinus lift,” “sinus augmentation,” “sinus grafting,” “sinus elevation” alone or in combination with “osteotome,” “dental implants,” “crestal,” and “transalveolar” using boolean operator “AND” and were chosen accordingly with previously published reviews [1, 5, 6]. Bibliographies of the selected articles were also manually searched.

Inclusion criteria for the studies were

- studies concerning osteotome-mediated sinus lifting procedure without using grafting materials;
- a minimum of 1-year followup after prosthetic rehabilitation;
- at least 20 patients treated;
- data on implant survival (SR) were reported.

Two authors (SC and MDF) independently screened abstracts and fulltext of the eligible articles for possible inclusion. In case of disagreement, a joint decision was taken by discussion.

Data from selected studies were extracted and recorded in a previously designed electronic form.

The fulltext of each included study was reviewed, and the following parameters were extracted:

- demographics of treated patients (age, gender, sample size);
- bone height (distance between bone crest and floor of the sinus);
- implant length;
- Implant survival rate;
- surgical or postsurgical complications;
- causes and occurrence of implant failure.

Weighted mean survival rate was calculated up to 5 years. The comparison between subgroups (follow-up duration, implant length, residual bone height) was made using Pearson's chi square test.

## 3. Results

The initial electronic search provided 438 items. After titles and abstracts screening, they are 361 articles were excluded because not pertinent with the aims of this paper. Of the 77 remaining articles, 62 were excluded because of not fulfilling the inclusion criteria. Fifteen articles were finally included in the analysis [12, 14–27].

Data about implant survival rates over time are presented in Table 1. It can be observed a great heterogeneity among studies regarding sample size (ranging from 20 to 983 patients) and study design. A total of 1767 implants were considered in this study. Survival rates were high in each considered follow-up time. The weighted mean survival was 98.02% one year after loading, 97.37% after 2 years, 97.47% after 3 years, and 96.77% after 5 years.

**Figure 1:**
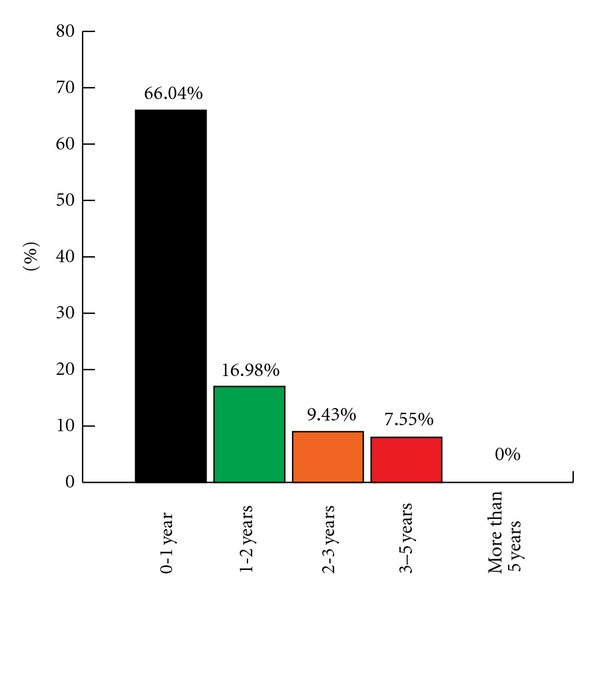
Failures distribution over time.

Two thirds (66.04%) of failures were recorded during the first year after loading as shown in Figure 1.

Implant length distribution in relation to implant survival at 1 year is shown in Table 2. Implant length varied among the studies, but it was greater than 10 mm in the majority of the considered studies. No correlation between implant length and survival rate could be demonstrated.

Alveolar bone height before and after surgical procedures is presented in Table 2. Mean residual bone height at baseline did not exceed 8.2 mm considering mean values. The higher mean bone height after surgery was 13.28 mm [27]. No correlation could be found between bone height and implant survival rate.

## 4. Technique Description and Case Report

**Figure 2:**
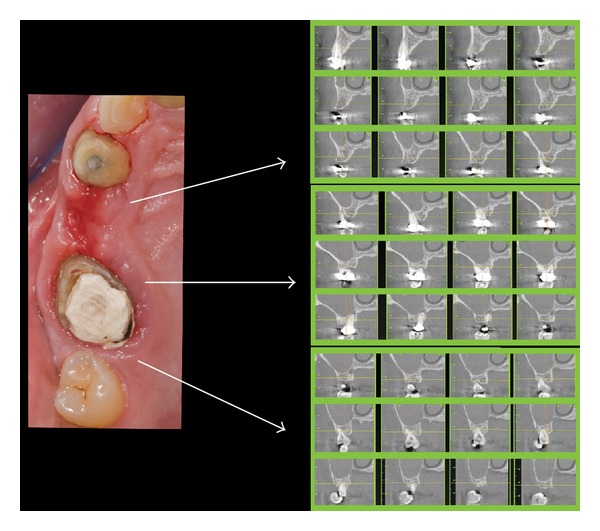
Clinical situation before surgery (clinical photo and TC sections).

A 45-year-old male patient, in general good health (ASA 1), nonsmoker, presented with a first left maxillary molar (2.6) exhibiting a destructive caries and referring vague, nonspecific symptoms. Radiographic examination revealed the presence of periradicular lesion of strictly endodontic origin, and a suitable restoration was considered unfeasible. In the same quadrant, the maxillary second premolar and second molar (2.5 and 2.7) were missing. Moreover, a tilted wisdom teeth (2.8) showed a lateral and vertical mobility associated with a pathological periodontal status (Figure 2). 

An experienced surgeon (ST) performed the entire surgical procedure.

### 4.1. Surgical Procedure

Preoperatively all patients rinsed with a 0.2% chlorhexidine solution for a minute as an antiseptic treatment in order to reduce the contamination of the surgical field. 

Patients' peripheral blood was collected using citrated tubes in order to prepare the platelet concentrate [28–30]. Briefly, the platelet concentrate is obtained by one-step centrifugation process (580 g for 8 minutes). The supernatant is then separated into two fractions paying care not to collect the leukocyte-rich layer: the deeper half is plasma very rich in growth factors (PVRGFs), and the upper half is plasma rich in growth factors (PRGFs). Each fraction is activated with calcium chloride a few minutes before use.

Local anaesthesia was administered with the use of articaine 4% and epinephrine 1 : 100.000.

A full thickness mucosal flap was raised, and the extraction of the mobilized teeth 2.6 and 2.8 was made with forceps in order to minimize the mechanical trauma to the surrounding bone. Implant surgical procedure was immediately performed after extraction of the involved teeth and accurate removal of the granulation tissue, when present, from the socket. 

**Figure 3:**
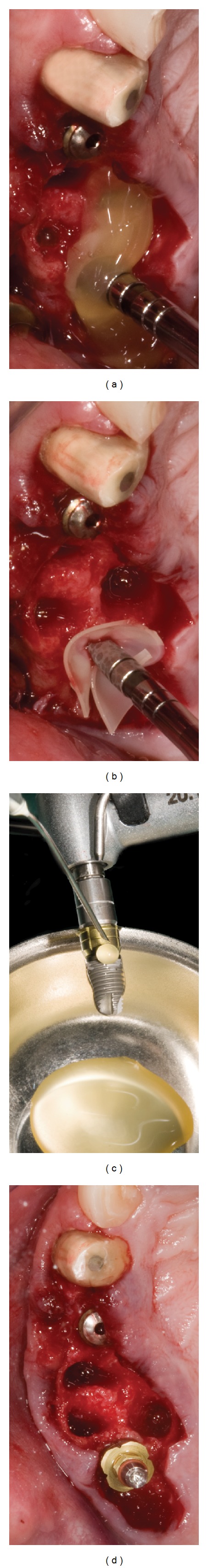
(a) Implant in site 1.5 was placed through standard protocol; a PRFG clot was positioned in the prepared socket before sinus floor elevation. (b) A membrane was placed apically in the so prepared site. (c) Before implant positioning, the fixture surface was bioactivated with liquid PRGF. (d) 2.5 and 2.7 implants in position.

**Figure 4:**
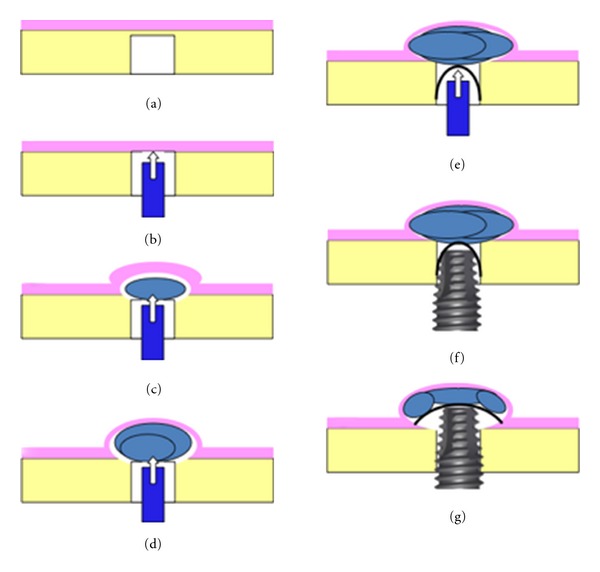
Schematic representation of osteotome-mediated sinus lift technique with the use of PRGF.

Three implants (BTI Biotechnology Institute, Alava, Spain) were placed. One was placed in the edentulous 2.5 site (Figure 3(a)), and implant installation was performed according to the protocol provided by the manufacturers. The other two implants were placed, respectively, in 2.6 post extraction site and in the bone bridge between 2.6 site and 2.8 site. In both sites, implant installation was performed using a modified technique of osteotome sinus floor elevation (OSFE) procedure [13] (Figure 4).

**Figure 5:**
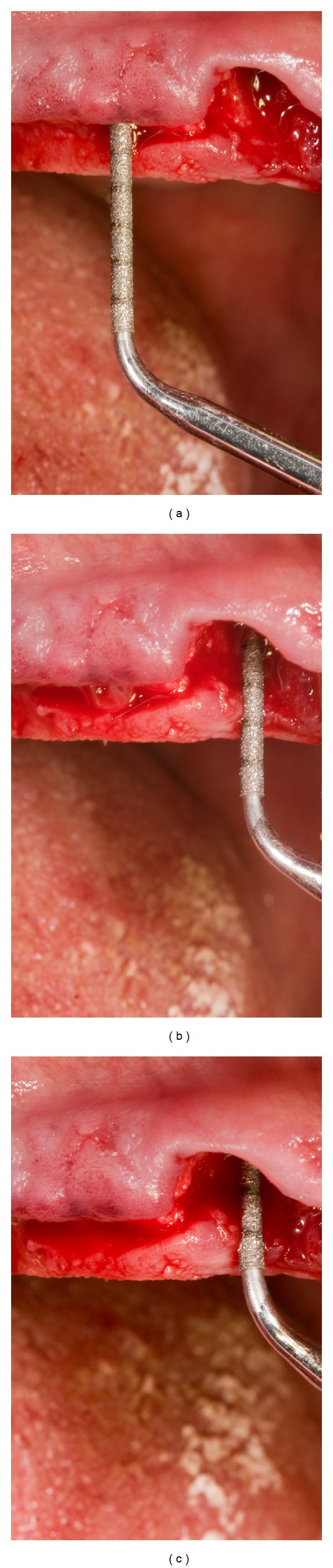
Use of piezoelectric inserts to prepare implant site.

Piezosurgical inserts (MB1, EMS, Nyon Switzerland) were used to prepare the implant sites until the Schneiderian membrane was reached (Figure 5). The sites depth was predetermined according to measurements obtained from the 3D radiographic examination. A Valsalva maneuver was done in order to detect the presence of an oroantral communication.

**Figure 6:**
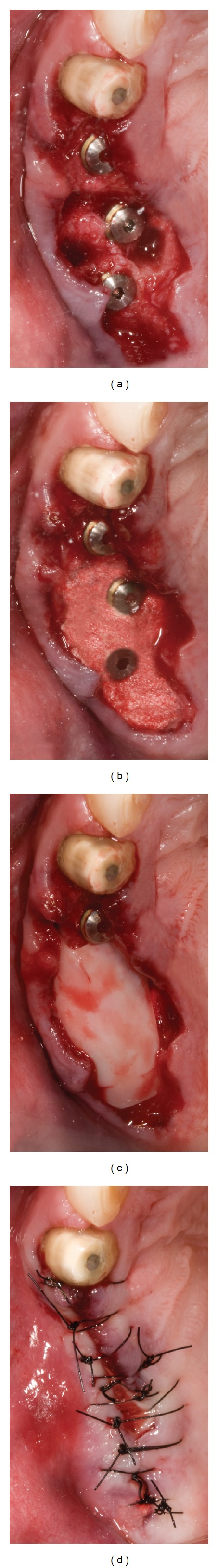
Gap filling and suture.

At this time, the sites were firstly embedded with liquid PVRGF (plasma very rich in growth factors) and subsequently a PRGF fibrin clot was gently pushed beyond the empty alveolus with the osteotome before raising the sinus floor (Figure 3(a)). The osteotome was used with minimal pressure and rotation and when necessary slight malleting to implode the sinus membrane in an apical direction (Figure 3(b)). After removing the osteotome, a Valsalva maneuver was done again. The osteotomy was to be underprepared by 1 mm relative to the final implants diameter to improve primary implant stability. The clot placement and the insertion of the osteotome were repeated several times until the required membrane lift was achieved; finally, a membrane of cross-linked collagen was placed in both sites (COVA, Biom'Up, Saint-Priest, France) (Figure 3(b)). The implant was embedded with PVRGF and inserted with a torque of at least 30 Ncm (Figures 3(c) and 3(d)). Three implants were placed: one 4.5 × 11.5 mm (2.5) and two 4 × 8.5 mm (2.6 and 2.7). A clot of PRGF combined with a biphasic and synthetic bone substitute, made by hydroxyapatite, calcium phosphate, and porcine-acellular collagen (Matribone, Biom'Up, Saint-Priest, France), was used as a gapfiller of the postextraction sockets (Figures 6(a) and Figure 6(b)). 

A cross-linked collagen membrane (COVA, Biom'Up, Saint-Priest, France) embedded with PVRGF was positioned over the cover screw (Figure 6(c)). The flaps were repositioned and secured with nonabsorbable silk 5-0 sutures (Ethicon Inc. Johnson & Johnson, Piscataway, NJ, USA). All implants were semisubmerged so that all parts of the defects were covered by mucosal tissue (Figure 6(d)).

After surgical phase, a standard pharmacological protocol was prescribed: amoxicillin 1 g every 8 hours for 5 days after surgery, nimesulide 100 mg twice daily for pain control if needed, and 0.2% chlorhexidine digluconate mouthwash twice daily for 1 week for plaque control. A soft diet was recommended, avoiding contact of the surgically involved zone with food for a few days if possible. Sutures were removed one week after surgery.

**Figure 7:**
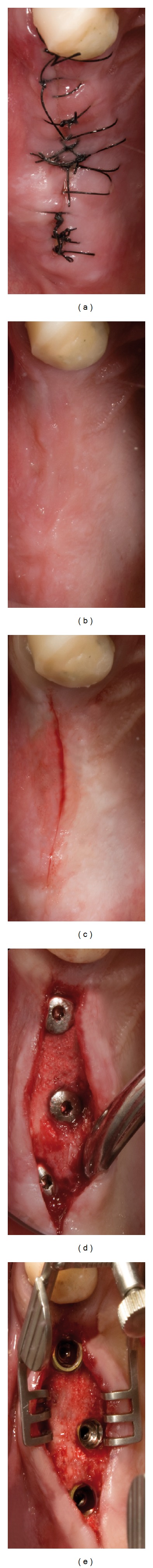
Second surgical phase.

**Figure 8:**
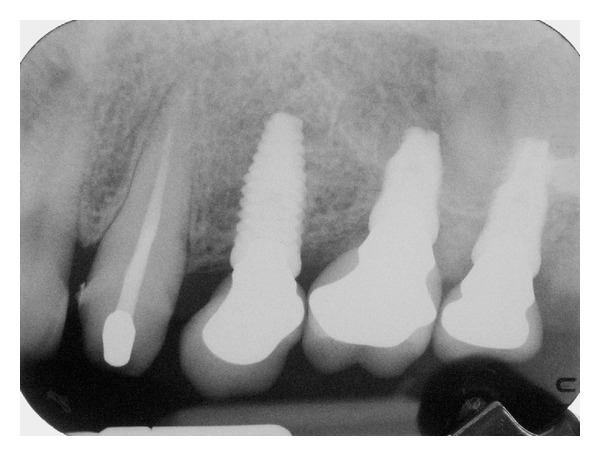
Radiographs taken at 6-month followup.

**Figure 9:**
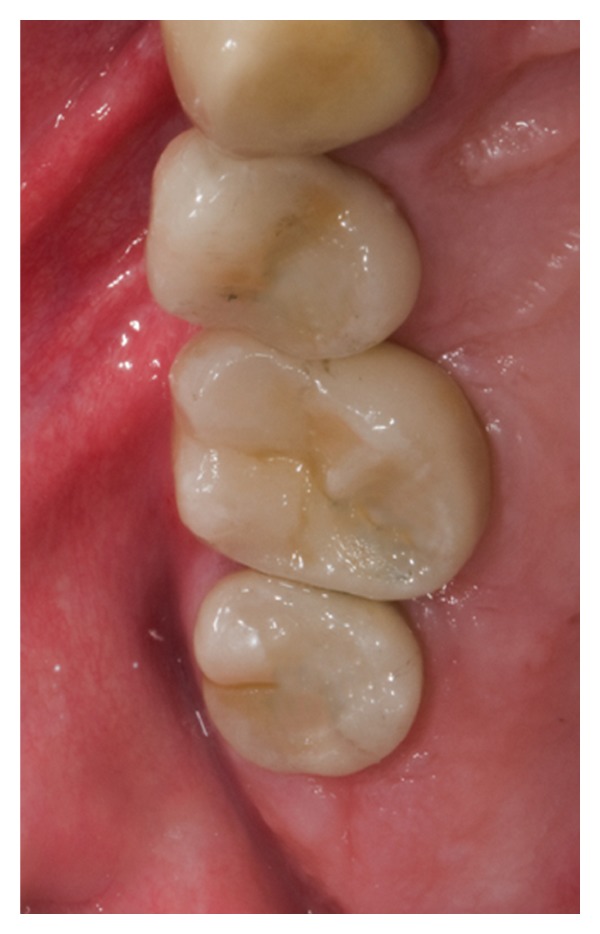
Occlusal view of the final prosthesis at 6-month followup.

After 3 months of healing, a surgical reentry procedure was performed. Full thickness flaps were elevated to access the marginal portion of the implant sites (Figure 7). The cover screws were replaced with healing caps and subsequently with permanent abutments, and the implants were loaded with the final restoration. The prosthesis was cemented (Figures 8 and 9). Complications were recorded any time they occurred.

### 4.2. Radiographic Evaluation

A standardized intraoral radiograph followed by a CBCT scan was taken before surgery (Figure 1).

Other intraoral periapical radiographs was taken immediately after implant placement, at the prosthetic phase, and at each follow-up visit (scheduled after 6 and 12 months of prosthesis function and yearly thereafter). 

Figure 9 is a radiograph taken at the 6-month followup. Radiographs were taken using a long cone paralleling technique and individual trays, in order to ensure reproducibility. Each periapical radiograph was scanned at 600 dpi with a scanner (Epson Expression 1680 Pro, Epson). 

### 4.3. Variables Assessed

Primary variables were (a) prosthesis success: prosthesis in function, without mobility. Prosthesis stability was tested by means of two opposing instruments' pressure. Prosthesis was considered as failed if its function was compromised for any reason; (b) implant success according to conventional criteria [31]; (c) postoperative quality of life based on the assessment of pain, swelling, general discomfort in the first week after surgery; (d) patient satisfaction for mastication function, phonetics, and aesthetics. The latter two variables were evaluated by means of questionnaires based on a five-point Likert scale [32]. 

Secondary variables were implant survival, the number and type of complications, mesial and distal changes of marginal bone level. 

## 5. Discussion

Osteotome-mediated sinus lifting technique has been demonstrated to be a viable alternative option in implant rehabilitation of atrophic posterior maxilla [4–6]. However, the advantage of the use of bone graft was not clearly shown in previous reviews [6].

The review of literature performed in the present paper confirmed that osteotome sinus lift technique performed without the adjunctive use of any bone substitutes is a safe and effective procedure.

The cumulative survival rates for implants placed in nongrafted sites are comparable with those placed in augmented grafted sites as was presented in previous systematic review [6].

The presented case report described implant placement in posterior atrophic maxilla. Osteotome sinus lifting technique was performed in a postextraction socket with the use of PRGF alone. Synthetic bone grafting material was used only to fill the gaps between implant and socket walls.

Crestal sinus lifting immediately after tooth extraction was described in few clinical reports [13, 33–35]. In the presented case report, a piezoelectric device was used in order to prepare implant site. Piezoelectric device allowed a more precise bone preparation of the socket walls where the use of standard drills could be complicated by the socket anatomy. Moreover, a piezoelectric preparation allowed the preservation of Schneiderian membrane in case of complete erosion of the sinus floor.

Platelet concentrate was used as an aid in membrane detachment acting as a cushion during the delicate use of osteotomes. The hydraulic pressure of PRGF clot caused a more controlled floor lifting avoiding excessive traumas to the cortical bone and to the Schneiderian membrane itself [12, 13, 36]. Furthermore, platelet derivatives can be beneficial to enhance soft tissue healing, reducing common postsurgical sequelae as swelling, pain, and hematoma [28, 29, 37]. This effect is achieved through the suppression of proinflammatory chemokines as IL-1 [38, 39] and through the release of many growth factors which promote tissue healing and regeneration [29]. 

In the presented case, a collagen membrane was then placed in contact with the PRGF clot, with the aim of guiding tissue regeneration in the apical portion. 

After implant placement, the gaps between the fixture and the socket walls were filled by a mixture of biphasic synthetic bone and PRGF liquid. The biphasic material gives a support to cellular adhesion and bone formation, but also a bioactivity that allows new bone formation [40, 41]. Moreover, it represented an ideal vehicle for PRGF growth factors and their release in the surrounding tissues [42].

The review of the scientific literature confirmed the successful outcomes of osteotome-mediated sinus lifting without the use of any bone substitute. This technique may be performed with the aid of platelet derivatives whose mechanical and biologic properties allow a safe detachment of the sinus membrane, possibly reducing the incidence of surgical and postsurgical complications.

The use of scaffold-like biomaterials to fill post-extraction sockets, when necessary, can emphasize the positive effect of platelet-derived factors, achieving an adequate bone filling, as shown in the present case report.

Although the presented technique may appear technically difficult, it showed a viable treatment option that could be considered and investigated through properly designed randomized controlled trials with adequate sample size.

## Figures and Tables

**Table 1:** Cumulative implant survival rates.

Study	*N*	*n*	1 *y*	*n*	2 *y*	*n*	3 *y*	*n*	5 *y*
Fermergård and Åstrand [14]	53	53	96,23			50	94,30		
Tetsch et al. [15]	983	983	98,88	887	97,88	805	98,39	529	97,83
Bruschi et al. [16]	66	66	95,45	63	95,45	63	95,45	63	95,45
Gabbert et al. [17]	92	92	95,65	83	95,65	83	95,65		
Jurisic et al. [18]	40	40	100,00	40	100,00	40	100,00		
Nedir et al. [19]	25	25	100,00	25	100,00	25	100,00		
Nedir et al. [20]	54	54	100,00						
Cavicchia et al. [21]	97	97	89,69	87	89,69	87	89,69	86	88,65
Diss et al. [12]	35	35	97,14						
Schmidlin et al. [22]	24	24	100,00	24	100,00				
Leblebicioglu et al. [23]	75	75	97,33	73	97,33				
Fugazzotto [24]	114	114	98,25	83	98,25	40	98,25		
Volpe et al. [25]	20	20	100,00						
Bruschi et al. [27]	68	68	100,00	68	100,00	68	100,00	68	100,00
Fornell et al. [26]	21	21	100,00						

*n* Total	1767	1767	98,02	1433	97,37	1261	97,47	746	96,77

**Table 2:** Bone height before and after surgery.

Study	Mean implant length	Mean ± SD (range) before surgery	Mean ± SD after surgery
Fermergård and Åstrand [14]	10,89	6,3 ± 0,3	10,7 ± 0,3
Tetsch et al. [15]	11,50	8,2	3,3
Bruschi et al. [16]	13,57	1–3	13,28 ± 1,23
Gabbert et al. [17]	10,29	NE	NE
Jurisic et al. [18]	10,72	NE	NE
Nedir et al. [19]	9,60	5,4 ± 2,3	10,3 ± 2,2
Nedir et al. [20]	8,37	2,5 ± 1,7	6,3 ± 1,5
Cavicchia et al. [21]	12,30	NE	NE
Diss et al. [12]	10,51	6,5 ± 1,7	9,8 ± 1,5
Schmidlin et al. [22]	8,60	5,0 ± 1,5	8,6 ± 1,3
Leblebicioglu et al. [23]	>11 mm	7 ± 1,3	10,9 ± 1,7
Fugazzotto [24]	9,16	NE	NE
Volpe et al. [25]	NR	7.2 ± 1.5	10.0 ± 1.0
Bruschi et al. [27]	13,50	6.02 ± 0,75	7.99 ± 1.16
Fornell et al. [26]	10,00	5.6 ± 2.1	8.6 ± 2.1
